# Evaluation of Tumor Cell Proliferation by Ki-67 Expression and Mitotic Count in Lymph Node Metastases from Breast Cancer

**DOI:** 10.1371/journal.pone.0150979

**Published:** 2016-03-08

**Authors:** Sura Aziz, Elisabeth Wik, Gøril Knutsvik, Tor Audun Klingen, Ying Chen, Benedicte Davidsen, Hans Aas, Turid Aas, Lars A. Akslen

**Affiliations:** 1 Centre for Cancer Biomarkers CCBIO, Department of Clinical Medicine, Section for Pathology, University of Bergen, Bergen, Norway; 2 Department of Pathology, Haukeland University Hospital, Bergen, Norway; 3 Department of Pathology, Vestfold Hospital Trust, Tønsberg, Norway; 4 Department of Pathology, Akerhus University Hospital, Lørenskog, Norway; 5 Department of Surgery, Haukeland University Hospital, Bergen, Norway; 6 Department of Surgery, Vestfold Hospital Trust, Tønsberg, Norway; University of Torino, ITALY

## Abstract

Few studies have addressed the risk of recurrence by assessing proliferation markers in lymph node metastasis from breast cancer. Here, we aimed to examine Ki-67 expression and mitotic count in lymph nodes in comparison with primary tumors. A cohort of node positive breast cancer (*n* = 168) was studied as a part of the prospective Norwegian Breast Cancer Screening Program (1996–2009). The percentage of Ki-67 positivity was counted per 500 tumor cells in hot-spot areas (x630). Mitotic count was conducted in the most cellular and mitotic active areas in 10 high power fields (x400). Our results showed that Ki-67 and mitotic count were significantly correlated between primary tumor and lymph nodes (Spearman`s correlation 0. 56 and 0.46, respectively) and were associated with most of the histologic features of the primary tumor. Univariate survival analysis (log-rank test) showed that high Ki-67 and mitotic count in the primary tumor and lymph node metastasis significantly predicted risk of recurrence. In multivariate analysis, mitotic count in the lymph node metastasis was an independent predictor of tumor recurrence. In conclusion, proliferation markers in lymph node metastases significantly predicted disease free survival in node positive breast cancer.

## Introduction

Breast cancer is a heterogeneous disease with complex molecular alterations [1]. Whereas known prognostic and predictive factors of the primary tumor (PT) are crucial in designing the best treatment plan and predicting clinical outcome [2–9], less is known about the significance of such factors examined in metastatic lesions, such as regional lymph nodes (LN) or at distant sites. As an example, the importance of tumor cell proliferation in primary tumor tissue, by mitotic count (MC) and Ki-67 expression has been extensively studied, but information about such markers in tumor metastases is very limited [10–18].

Recently, an emerging interest for identifying additional prognostic and predictive factors by studying biological markers in metastatic tumor tissues has occurred. For instance, a number of studies have addressed the prognostic impact of metastatic tumor size and tumor burden in axillary LN [19–22], while others reported the proliferation and molecular subtype of breast cancer in LN metastasis with partially conflicting results [23–27]. Furthermore, the prognostic role of Ki-67 in LN and relapse biopsies has been studied with different methods [23, 25, 28, 29]. Some have reported the change in Ki-67 expression from low in PT into high in metastasis as predictive for poor post-relapse survival [23, 28]. Additionally, there are no studies showing the potential impact of mitotic count in LN metastasis on survival and its correlation with characteristics of the primary tumor.

Here, we investigated the prognostic significance of tumor cell proliferation, by Ki-67 expression and mitotic count, in axillary LN metastasis and their correlation with known prognostic features of the primary tumors. Furthermore, we aimed to identify the most high-risk subgroup which could potentially be of interest in treatment stratification, based on the matched proliferation pattern in primary tumors and LN metastasis.

## Material and Methods

### Patient Series

The cohort *(n* = 816) represents women diagnosed with primary invasive breast cancer (mean age 59 years, range 50–69) who resided in two counties in Norway (Hordaland, Vestfold) and participated in the prospective population-based Norwegian Breast Cancer Screening Program during 1996–2009 [10, 30, 31]. Hordaland and Vestfold counties have approximately 730,000 inhabitants, this represents about 15% of the total population in Norway. Written informed consent was not obtained from the patients, but in accordance to the national ethical guidelines for such retrospective studies, all participants were contacted with written information on the study and asked to respond if they objected. A subgroup of 231 cases had LN metastasis (S1 Fig, S1 Table). Key inclusion criteria were: 1: the diagnosis was made by histologic examination of LN specimens (sentinel node or axillary dissection); 2: the metastatic lesion examined should be equal to or larger than 2 mm in diameter, to ensure sufficient amount of tumor tissue for mitotic count and assessment of Ki-67 expression. Finally, 168 cases were included for the study of proliferation markers Ki67 and mitotic count. Complete clinical follow-up information was obtained from the medical journals (last date of follow-up was June 1, 2015 for Hordaland, and Jan 1, 2015 for Vestfold). Outcome data include survival time, time to first distant metastasis, and cause of death. During the follow up, 36/168 (21%) died of breast cancer, 13/168 (8%) died of other causes, whereas 28/168 (17%) are still alive with metastatic breast cancer, and 91 (54%) were alive without local or distant recurrence. Altogether, there were 60 patients (36%) who developed distant metastases, either at one site in 31 cases (52%), or multiple locations in 29 cases (48%). The median follow-up for the survivors was 90 months.

Our study was approved by the Western Regional Committee for Medical and Health Research Ethics, REC West (REK 2014/1984), REK South-East (REK 2008/16904).

### Specimen Characteristics

Samples from tumor tissues were obtained at the time of surgery. Fixation of tumor specimens followed standard protocols, using 10% buffered formalin for a range of 1–13 days. After processing and paraffin embedding, 4–5 μm sections were cut and mounted on poly-lysine coated glasses. Storage time of the sections was no longer than 14 days at 4°C until staining with Ki-67 was performed. Storage of the archival samples was up to 19 years.

Features of the primary tumor at time of diagnosis, including tumor diameter, histologic type, histologic grade, lymph node status, hormone receptor status, HER2 status, molecular subtype and data on national guidelines for treatment were recorded (S2 Table) [10]. Medical journals were retrieved to collect data on the extent of distant metastasis and the time for developing first distant metastasis based on clinical and radiological staging. The molecular subtype was determined according to the recommendations from St. Gallen 2013 [8] with minor modifications; the cut-off point for ER and PR positivity was 10% according to our national guidelines at that time. Notably, six cases (2.5%), which were negative for ER and Her2 status while positive for PR status, were regarded as luminal. Categorizing histologic grade into two groups (grade 1–2 versus grade 3) was in accordance with treatment guidelines by the Norwegian Breast Cancer Group (NBCG) [32]. Out of 229 cases (99%) with available information about surgical procedure, 101(44%) received sentinel node dissection followed by axillary node surgery, with a median number of 1 metastatic node (range 1–19), while axillary node dissection was performed in 122 cases (53%), with median number of 2 metastatic nodes (range 1–13). Six cases (2.6%) had only sentinel node dissection due to the small size of the metastasis (≤ 0.2 mm). Median number of lymph nodes removed was 12 (range 1–26 nodes) in cases with sentinel node dissection followed by further axillary dissection, while median of lymph nodes removed in cases with only axillary dissection was 11 (range 1–29 nodes), and 3 (range 1–10 nodes) in cases with only sentinel node removal

#### Mitotic count

The section with the largest focus of lymph node metastasis (≥ 2.0 mm) was examined, and the most cellular area with the highest number of mitotic figures (hot-spot) was marked for counting. Mitotic figures were counted in 10 consecutive HPFs (x400), and finally recorded per mm². In 4 cases (2%), mitotic figures were counted in less than 10 HPF (5–8 HPF) because of small metastases. Caution was taken for areas with necrosis, fibrosis, and numerous lymphocytes. Similarly, assessment of MC in PT was done by the same observer (S.A.). The inter-observer variability was tested on 50 cases showed a high correlation (Kappa value 0.84, *P* < 0.001; Spearman`s correlation coefficient 0.78, *P* < 0.001). Intra-observer variability was evaluated by randomly selecting 20 cases for assessment of MC after a period of 5 months with excellent correlation between the two reads (Kappa value 0.99, *P* < 0.001; Spearman`s correlation coefficient 0.92, *P* < 0.001).

#### Ki-67 immunostaining

Immunohistochemistry was performed on 4–5μm standard sections of formalin fixed, paraffin embedded tumor tissue from the largest LN metastases using the same protocol as for primary tumors[10]. Briefly, de-waxing with xylene/ethanol at different concentrations (100%, 96% and then 80%) was first performed before retrieval in a pressure cooker (Decloaking Chamber Plus, Biocare Medical). Staining procedures were performed by the Dako autostainer using K4061/ Envision Dual Link System (rabbit and mouse). We incubated sections for 30 minutes at room temperature with monoclonal rabbit antibody (M 7240, cloneMIB-1, DAKO) at 1:100 dilutions. Diaminobenzidine (DAB) as chromogen was added for 10 min, then hematoxylin and eosin (H&E) as a counterstain for 3 minutes. Sections from tonsils were used as positive controls. Negative controls were obtained by replacing the primary antibody with Tris-buffered saline. Positive and negative controls were included in each run.

#### Evaluation of Ki-67 staining

Slides were examined for Ki-67 labelling index by one pathologist (S.A.), blinded to patient outcome and the previous Ki-67 assessment in the PT. Using light microscopy (Leica DMLB) with an eye piece grid for counting at x630 magnification (field area 0.238 mm²), we followed the approach used by Weidner et al. [33]. The slides were first scanned at x100 for selecting the area with highest density of Ki-67 labelled cells. Such areas were marked as a hot-spot, and only nuclei crossing the horizontal lines of the grid were counted. Tumor cells showing a distinct nuclear staining, strong or weak, were counted. Cells with staining only in the cytoplasm without any clear nuclear staining were not considered positive. Finally, the percentage of Ki-67 labelled tumor cells was calculated. Some caution was taken in avoiding certain areas as described for MC. Using the same procedure, assessment of Ki-67 index in PT was performed by the same pathologist (S.A.) on the previously stained slides [10] with good inter-observer agreement (Kappa value 0.70, *P* <0.001 for PT; Spearman`s correlation coefficient 0.95, *P* < 0.001). Intra-observer variability for assessment of Ki-67 was evaluated in 20 cases after a period of 5 months with excellent correlation between the two reads (Kappa value 0.99, *P* <0.001, Spearman`s correlation coefficient 0.92, *P* <0.001).

#### Determination of cut-off values for Ki-67 and MC

Continuous variables were categorized by either median value (Ki-67: 14.2%, 14.6%), or quartile limits (MC: 5.4 /mm², 4.2 /mm²) for PT and LN, respectively, with consideration of the frequency distribution plot for each marker, the number of events in subgroups and their survival patterns. A cut-off value of 20% in Ki-67 was used as a reference to St. Gallen 2013[8].

#### Statistical analysis

Data were analyzed using SPSS (Statistical Package of Social Sciences), Version 22.0 (SPSS. Inc. Chicago, IL). A two sided *P* value less than 0.05 was considered to be statistically significant. Categories were compared using Pearson`s or Fisher`s Exact tests when appropriate. Mann-Whitney U test was used to compare continuous variables between groups. Wilcoxon Signed Rank test was used to compare related samples. Non-parametric correlation was tested by Spearman’s rank correlation, and Kappa statistics was used to test inter- and intra-observer agreement. For proliferation markers, concordance was defined as either high or low in both sites (PT and LN metastases), while discordance was defined as high at one site and low at the other.

For survival analysis, the end-point was disease free survival (DFS) which is defined as the time in months from the date of diagnosis to the date of developing the first distant metastasis. Univariate survival analysis was performed using log-rank test to compare differences in survival time between categories. Patients who did not develop distant metastasis were censored in the analyses of disease free survival. The influence of co-variates on disease free survival was analyzed by Cox`s proportional hazards method and tested by backward stepwise likelihood ratio. All variables were tested by log-minus-log plot to determine their ability to be incorporated in a multivariate analysis. For vector graphics, Adobe illustrator CS6 was used to edit the figures.

## Results

### Ki-67 in Primary Tumors and Lymph Node Metastasis

**Fig 1:**
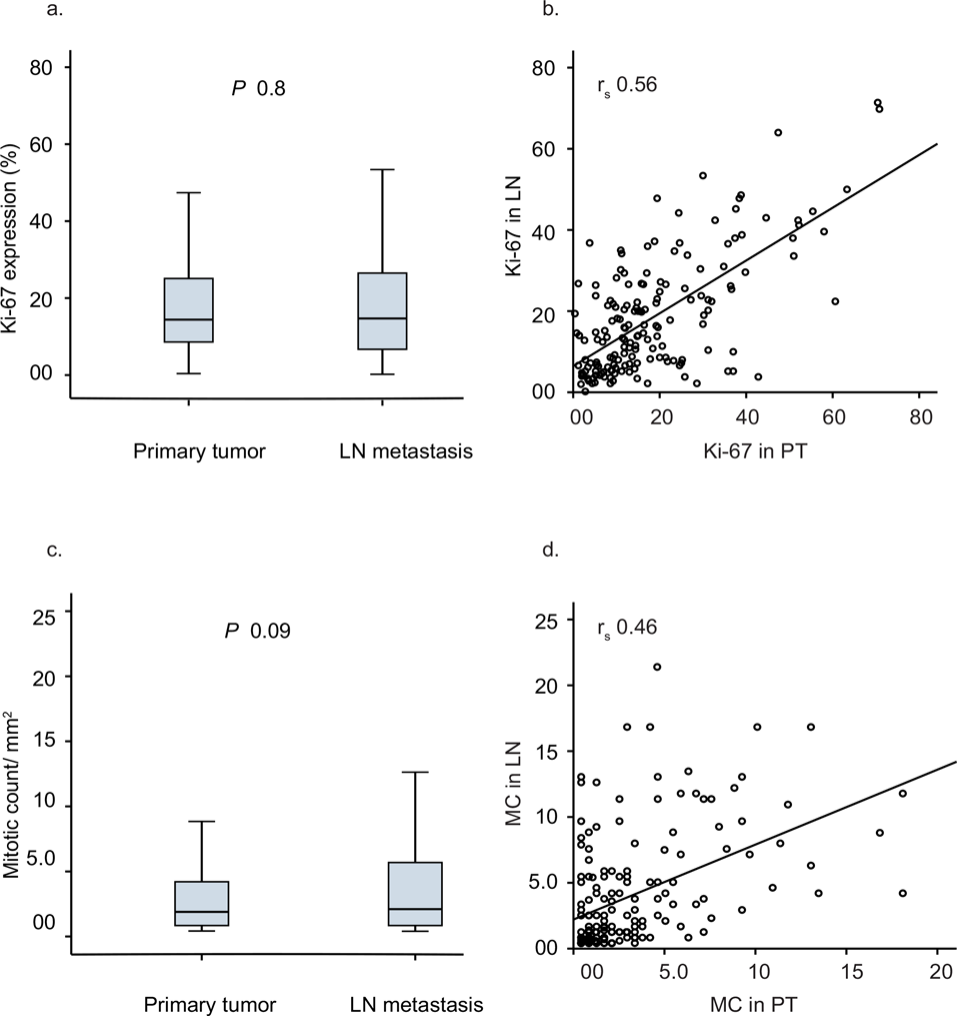
Proliferation markers in primary tumors and lymph node metastases. Box plots (a. and c.) Horizontal lines inside the boxes represent the median values; box limits indicate the 25^th^ and 75^th^ percentiles. Scatter plot (b. and d.) for % Ki-67 and MC in PT and LN metastasis.

Ki-67 values in PT and in LN metastasis showed no significant difference (median 14.2%, 14.6%, respectively, *P* 0.8, Wilcoxon Signed Rank test) (Fig 1A) (S2A Fig). (Spearman’s correlation 0.56, *P* <0.001) (Fig 1B). High Ki-67 was significantly associated with unfavorable features of the primary tumors (Table 1).

**Table 1:** Ki-67 expression and histopathologic features of the primary tumors.(*n* = 168).

Variable	Ki-67 LN		*P* value ^d^	Ki-67 PT		*P* value ^d^
	N	(Median)		N	(Median)	
**Tumor diameter** ^a^			0.050			0.003
≤ 2 cm	95	(13.4%)		95	(11.8%)	
> 2 cm	73	(18.2%)		73	(19.2%)	
**Histologic grade**			<0.001			<0.001
Grade 1–2	129	(11.4%)		129	(11.8%)	
Grade 3	39	(33.6%)		39	(30.0%)	
**No. of positive nodes** *			0.016			NS
1–3 nodes	107	(12.2%)		107	(13.2%)	
≥ 4 nodes	60	(20.2%)		60	(14.8%)	
**MC/mm² (PT)** ^b^			<0.001			<0.001
≤ 4.2	127	(11.5%)		127	(12.8%)	
˃ 4.2	41	(30.4%)		41	(29.4%)	
**ER**			<0.001			0.001
Positive	141	(13.0%)		141	(13.0%)	
Negative	27	(29.4%)		27	(20.2%)	
**PR**			<0.001			0.016
Positive	115	(11.2%)		115	(13.0%)	
Negative	53	(22.0%)		53	(19.2%)	
**HER2 status**			<0.001			<0.001
Negative	143	(13.0%)		143	(12.8%)	
Positive	25	(25.6%)		25	(24.6%)	
**Molecular subtype** ^c^			<0.001			<0.001
Luminal A	62	(8.0%)		62	(8.5%)	
Luminal B/ HER2 -	69	(16.8%)		69	(19.2%)	
Luminal B / HER2+	15	(20.2%)		15	(24.6%)	
HER2 +	11	(33.8%)		11	(23.4%)	
Triple negative	11	(38.0%)		11	(38.8%)	
**Distant metastasis**			<0.001			<0.001
No	108	(12.3%)		108	(11.8%)	
Yes	60	(20.9%)		60	(19.5%)	

N, number of cases in each subgroup; Median, median of Ki-67 in each subgroup

LN, Lymph node; PT, Primary tumor; MC, mitotic count; ER, estrogen receptor;

PR, progesterone receptor; NS, not significant

^a^ Cut-off value by median

^b^ Cut-off value by upper quartile

^c^ Molecular subtype according to St. Gallen 2013

^d^ Mann-Whitney U test

* One case was missing with respect to number of lymph nodes involved

Further, we stratified the combined dichotomized Ki-67 in PT and LN metastases into four subgroups, using median values as cut-off; high Ki-67 in both (termed HH, *n* = 58, 35%); high Ki-67 in PT and low in LN (termed HL, *n* = 27, 16%); low Ki-67 in PT and high in LN (termed LH, *n* = 26, 15%); low Ki-67 in both tumor sites (termed LL, *n* = 57, 34%) (S3–S6 Figs). In reference to the Ki-67 cut-off value recommended in the St Gallen 2013 guidelines [12], we noticed that some cases (17%) showed an increase of their Ki-67 value from ≤ 20% in the primary tumor to > 20% in their LN metastasis (termed as LH by 20% cut-off point). This subgroup (LH) had 9 of 28 cases (32%) representing luminal A-like primary tumors (using 20% in distinguishing between luminal A and luminal B subtype).

#### Mitotic Count in Primary Tumors and Lymph Node Metastasis

Median value for mitotic count in primary tumor was slightly lower compared to their matched LN metastases (1.8 vs 2.1 mitotic figure/mm², respectively, *P* 0.09, Wilcoxon Signed Rank test) (Fig 1C) (S2B Fig). The values in PT and matched LN metastases were significantly correlated (Spearman’s correlation 0.46, *P* 0.001) (Fig 1D). High MC was significantly associated with aggressive features of the primary tumors (Table 2).

**Table 2:** Mitotic count (MC) and clinico-pathologic features of the primary tumors (*n* = 168).

Variable	MC LN		*P* value ^c^	MC PT		*P* value ^c^
	N	(Median)		N	(Median)	
**Tumor diameter ^a^**			NS			NS
≤ 2 cm	95	(1.68)		95	(1.68)	
> 2 cm	73	(2.52)		73	(2.10)	
**Histologic grade**			<0.001			<0.001
Grade 1–2	129	(1.38)		129	(1.68)	
Grade 3	39	(7.57)		39	(5.47)	
**No. of positive nodes** *			0.044			NS
1–3 nodes	107	(1.68)		107	(2.10)	
≥ 4 nodes	60	(2.73)		60	(1.68)	
**ER**			0.003			0.010
Positive	141	(1.68)		141	(1.68)	
Negative	27	(5.05)		27	(4.60)	
**PR**			0.003			0.002
Positive	115	(1.60)		115	(1.68)	
Negative	53	(3.78)		53	(3.36)	
**HER2 status**			0.006			NS
Negative	143	(1.68)		143	(1.68)	
Positive	25	(5.47)		25	(2.25)	
**Ki-67 PT (%)** ^a^			<0.001			<0.001
≤ 14.2%	83	(1.26)		83	(1.26)	
˃ 14.2%	85	(3.78)		85	(2.52)	
**Molecular subtype** ^b^			0.001			0.003
Luminal A	62	(1.26)		62	(1.26)	
Luminal B/ HER2 -	69	(1.68)		69	(2.10)	
Luminal B / HER2+	15	(5.47)		15	(1.68)	
HER2 +	11	(5.05)		11	(5.05)	
Triple negative	11	(8.83)		11	(5.47)	
**Distant metastasis**			<0.001			NS
No	108	(1.26)		108	(1.68)	
Yes	60	(3.99)		60	(2.31)	

N, number of cases in each subgroup; Median, median of MC in each subgroup

LN, Lymph node; PT, Primary tumor; ER, estrogen receptor; PR, progesterone receptor;

NS, not significant

^a^ Cut-off point was median

^b^ Molecular subtype according to St. Gallen 2013

^c^ Mann-Whitney U test

* One case was missing with respect to number of lymph nodes involved

As for Ki-67 expression, the combined mitotic count in PT and LN metastases was used to generate four subgroups of proliferation using the upper quartile as cut-off point. These subgroups were either concordant high (HH, *n* = 24, 14%), discordant (HL, *n* = 17, 10%, and LH, *n* = 18, 11%), or concordant low MC (LL, *n* = 109, 65%) (S3–S6 Figs).

#### Proliferation Markers and Patient Outcome

**Fig 2:**
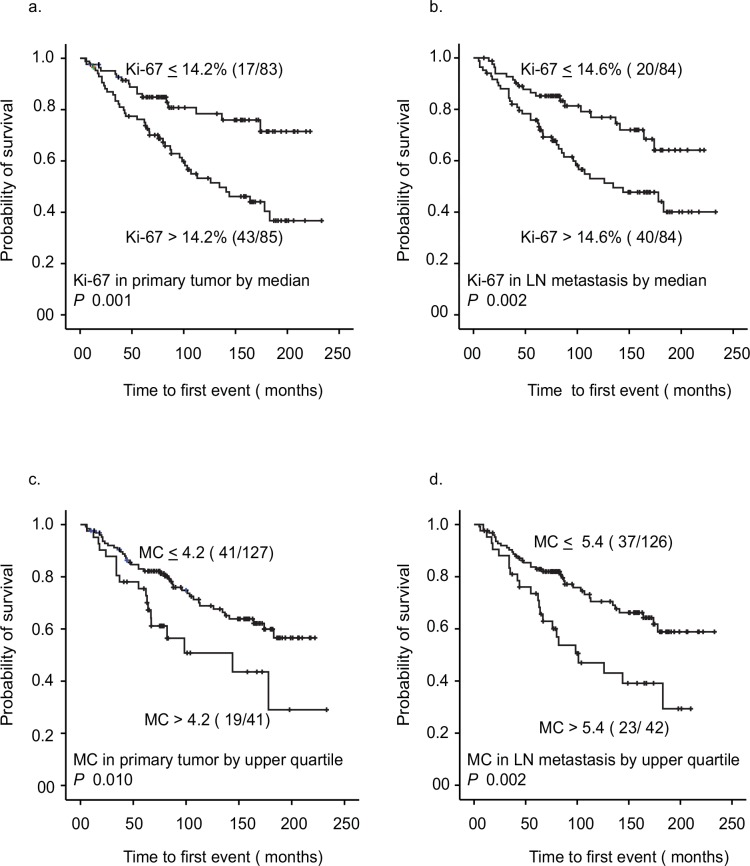
Survival analysis for Ki-67 and mitotic count in primary tumors and LN metastases. Survival curves (Kaplan-Meier method) are shown for Ki-67 in PT and LN metastasis (a. and b.), and MC for PT and LN metastasis (c. and d.). Number of events / number of cases are given in parenthesis.

Univariate survival analysis showed that Ki-67 in LN metastasis and PT were both predictive for disease recurrence (Fig 2A and 2B). Subgroups with combined proliferation (HH, LH, and HL) demonstrated a reduced DFS with a significant difference between these subgroups combined and patients with concordant low proliferation (LL) (S7A Fig). Similar results were seen for mitotic count in LN metastasis and PT separately (Fig 2C and 2D), and as a combined variable (S7B Fig). Univariate survival analysis by Cox proportional hazards method is illustrated in Tables 3 and 4.

**Table 3:** Univariate survival analysis of features of primary tumors (*n* = 168).

Variable	HR	95% CI	*P* value ^c^
**Tumor diameter** ^a^			0.004
≤ 2 cm	1		
>2 cm	2.1	1.2–3.5	
**Histologic grade**			<0.001
Grade 1–2	1		
Grade 3	2.8	1.6–4.7	
**No. of positive nodes**			0.006
1–3 nodes	1		
≥ 4 nodes	2.0	1.2–3.3	
**ER**			0.010
Positive	1		
Negative	2.1	1.1–3.8	
**PR**			0.001
Positive	1		
Negative	2.3	1.4–3.9	
**HER2 status**			0.001
Negative	1		
Positive	2.6	1.4–4.6	
**Molecular subtype** ^b^			<0.001
Luminal A	1		
Luminal B/ HER2 -	2.5	1.2–5.3	
Luminal B / HER2+	4.0	1.6–9.9	
HER2 +	6.7	2.6–17.2	
Triple negative	4.6	1.6–12.8	

HR, Hazards ratio; CI, confidence interval; ER, estrogen receptor;

PR, progesterone receptor; NS, not significant

^a^ Cut-off value by median

^b^ Molecular subtype according to St. Gallen 2013

^c^ P value calculated by Cox`s proportional hazards method

**Table 4:** Univariate survival analysis of proliferation markers examined in lymph node metastases (*n* = 168).

Variable	HR	95% CI	*P* value ^c^
**Ki-67% (PT)** ^a^			0.001
≤ 14.2	1		
˃ 14.2	2.5	1.4–4.5	
**Ki-67% (LN)** ^a^			0.005
≤ 14.6	1		
˃ 14.6	2.1	1.2–3.6	
**MC/mm² (PT)** ^b^			0.010
≤ 4.2	1		
˃ 4.2	2.0	1.1–3.5	
**MC/mm² (LN)** ^b^			0.003
≤ 5.4	1		
˃ 5.4	2.2	1.3–3.7	

HR, Hazards ratio; CI, confidence interval; MC, mitotic count; PT, primary tumor; LN, lymph node

^a^ Cut-off value by median

^b^ Cut-off value by upper quartile

^c^ P value calculated by Cox`s proportional hazards method

In multivariate analysis, after including tumor diameter, histologic grade, number of positive nodes, and both proliferation markers in one model, only mitotic count in LN metastasis was an independent predictor for disease free survival (Table 5).

**Table 5:** Multivariate survival analysis (Cox`s proportional hazards method) Final model after initial inclusion of tumor diameter, histologic grade, Ki-67 in PT ^b^, Ki-67 in LN ^b^, MC in PT ^b^, MC in LN ^b^ (*n* = 168).

Variable	HR	95% CI	*P* value ^c^
**Tumor diameter** ^a^			
≤ 2 cm	1		
>2 cm	1.79	1.06–3.03	0.028
**Histologic grade**			
Grade 1–2	1		
Grade 3	1.87	1.04–3.36	0.034
**MC/mm² (LN)** ^b^	1.06	1.00–1.12	0.024

HR, Hazards ratio; CI, confidence interval

PT, primary tumor; LN, lymph node; MC, mitotic count

^a^ Cut-off by median value

^b^ Continuous variable

^c^ Likelihood ratio

## Discussion

Recently, increasing focus has been put on the assessment of prognostic and predictive biomarkers in tissue samples from metastatic lesions for treatment decisions and to account for tumor evolution [5]. Breast cancer studies have attempted to examine differences between primary tumors and LN metastases for hormone receptors, HER2 status and Ki-67 [23, 25, 26, 29, 34–39]. For tumor cell proliferation, such studies are limited and not conclusive. Here, we asked whether examination of Ki-67 expression and mitotic count in LN metastases could add prognostic information and potential predictive value above that provided by the primary tumor alone. By using a population-based cohort derived from the prospective Norwegian Breast Cancer Screening Program, we found that high Ki-67 and MC in primary tumors as well as LN metastasis significantly predicted the risk of tumor recurrence. As a novel finding, the prognostic power of MC in LN metastasis was independently significant. To the best of our knowledge, our study is one the largest series to explore the prognostic impact of mitotic count in LN metastasis [40, 41].

Mitotic count and Ki-67 were positively correlated between the primary tumors and matched LN metastases, whereas the median values showed only limited differences between the two sites for both markers. For mitotic count, when using the upper quartile as cut-off value, we observed that 21% of the cases showed a discordant pattern when comparing PT and LN metastasis, with 11% of all changing from low proliferation in the PT to high level in LN metastasis. The clinical consequence of such a pattern is currently not known but must be considered. From a prognostic point of view, we found an independent value of adding mitotic count from the lymph node metastatic lesion.

Ki-67 expression in LN metastasis showed slightly but not significantly higher values than in their PT, in line with other studies [23, 26, 28, 29]. Significant correlations with known unfavorable features in PT was found, in contrast to a previous study [23]. Similar to mitotic count, 32% of the cases showed a discordant pattern when comparing PT and their LN metastases (by median value), and 15% of all cases changed from low to high proliferation when metastasized. Again, it should be considered whether such a pattern could have a potential clinical consequence in the setting of adjuvant therapy. For instance, discordant patterns of other biomarkers, like ER, PR and HER2, result in changed treatment according to their expression at the metastatic site [39, 42].

Considering Ki-67 and MC in matched pairs of PT and LN metastasis and time to recurrence, we demonstrated an increased risk of recurrence when high proliferation was detected at least at one site (PT or LN) or both. Of particular importance, 15% of cases examined with Ki-67 and 11% of the cases evaluated by mitotic count changed their proliferation values from low in primary tumor into high in LN metastases, and this could have a potential clinical impact. Interestingly, when using 20% as a cut-off point for Ki-67 in the adjuvant setting, as currently recommended for primary tumors according to St. Gallen guidelines in 2013, 28 cases (17%) changed from low to high proliferation. It should be considered whether these cases would benefit from adjuvant treatment as usually based on Ki-67 from the primary lesion. Further studies are therefore necessary to clarify this issue.

The present findings support the view of heterogeneity between primary tumors and lymph node metastasis with respect to proliferation markers that might be important in treatment decisions. In conclusion, our data support a potential value of proliferation markers determined in lymph node metastasis, as a complementary procedure to define high-risk groups that need individualized treatment. Future studies are necessary to validate our findings.

## Supporting Information

S1 Fig Flow diagram. Flow diagram for the cases included in this study. Abbreviations: SN; Sentinel node, AXLD; Axillary node dissection, FNAC; Fine Needle Aspiration Cytology, CNB; Core Needle Biopsy, n; number of cases.
(PDF)

S2 Fig The difference in Ki-67 expression and mitotic count between PT and LN. A parallel graphic illustration for the difference in ki-67 expression and mitotic count between PT and LN metastases.
(PDF)

S3 Fig High mitotic count and Ki-67 in PT and LN. High mitotic count (asterisk) in PT (A) and LN (B). High Ki-67 expression in PT (C) and LN (D) (Leica, x 400).
(PDF)

S4 Fig Low mitotic count and Ki-67 in PT and LN. Low mitotic count (aterisk) in PT (A) and LN (B). Low Ki-67 expression in PT (C) and LN (D) (Leica, x 400).
(PDF)

S5 Fig Low mitotic count and Ki-67 in PT and high in LN. Low mitotic count (asterisk) and low Ki-67 expression in PT (A, C, respectively), while high mitotic count (asterisk) and high Ki-67 expression in LN (B, D, respectively) (Leica, x 400).
(PDF)

S6 Fig High mitotic count and Ki-67 expression in PT and low in LN. High mitotic count (asterisk) and high Ki-67 expression in PT (A, C, respectively), while low mitotic count (asterisk) and low Ki-67 expression in LN (B, D, respectively) (Leica, x 400).
(PDF)

S7 Fig Survival curves for Ki-67 and MC in matched pairs. Survival curves (Kaplan-Meier method) are shown for Ki-67 (a) and for MC (b) in PT and LN metastasis in matched pairs. Number of events / number of cases are given in parenthesis.
(PDF)

S1 Table Clinico-pathologic characteristics of primary tumors for the whole cohort. Clinico-pathologic characteristics of primary tumors for the whole cohort before exclusion of some cases (n = 231).
(DOCX)

S2 Table Clinico-pathologic characteristics of primary tumors for the study cohort. Clinico-pathologic characteristics of primary tumors for the cases which were finally included in this study (*n* = 168).
(DOCX)

S3 Table Data-sheet for cases used in this study. (XLSX)

## Abbreviations

H&E: Hematoxylin & Eosin
PT: Primary tumor
LN: Lymph node
HR: Hazards ratio
CI: Confidence interval
MC: Mitotic count
ER: Estrogen receptor
PR: Progesterone receptor
HER2: Human epidermal growth factor 2
TNBC: Triple negative breast cancer
CNB: Core needle biopsy
DFS: Disease free survival
Met: Metastases
N: Number of cases
AXND: Axillary node dissection
SN: Sentinel node
HPF: High power field
