# Supplementary material for: Evaluation of Tumor Cell Proliferation by Ki-67 Expression and Mitotic Count in Lymph Node Metastases from Breast Cancer
Source: PLoS One. 2016 Mar 8;11(3):e0150979. doi: 10.1371/journal.pone.0150979 (PMC4783103; doi:10.1371/journal.pone.0150979)
Supplement: S1 Fig — Flow diagram for the cases included in this study. Abbreviations: SN; Sentinel node, AXLD; Axillary node dissection, FNAC; Fine Needle Aspiration Cytology, CNB; Core Needle Biopsy, n; number of cases. (PDF) [file pone.0150979.s001.pdf]

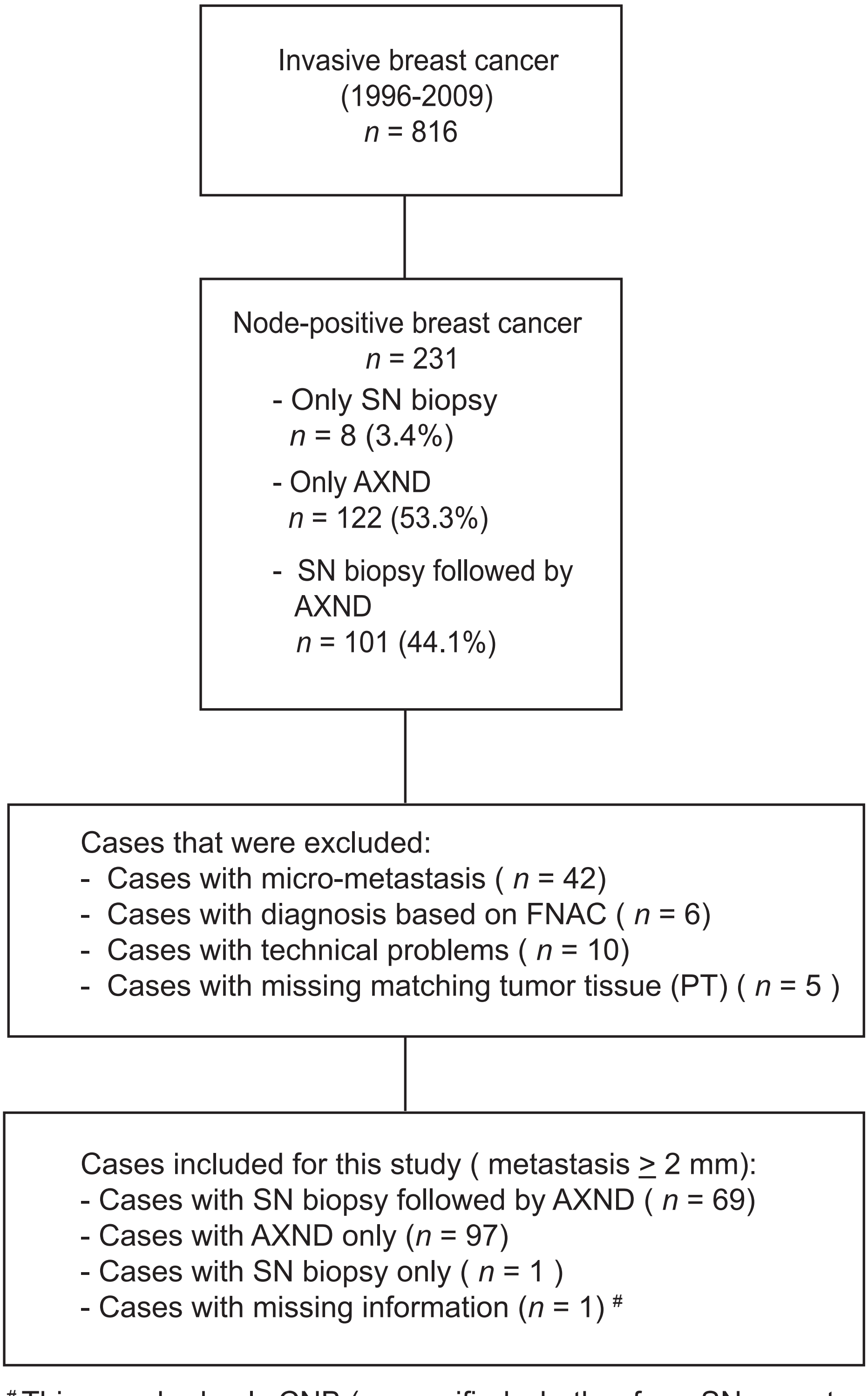

This case had only CNB (unspecified whether from SN or rest of axillary nodes) performed as a surgical approach because of the deteriorated clinical condition of the patient.
