# Supplementary figures and images for: Evaluation of Tumor Cell Proliferation by Ki-67 Expression and Mitotic Count in Lymph Node Metastases from Breast Cancer
Source: PLoS One. 2016 Mar 8;11(3):e0150979. doi: 10.1371/journal.pone.0150979 (PMC4783103; doi:10.1371/journal.pone.0150979)

a.

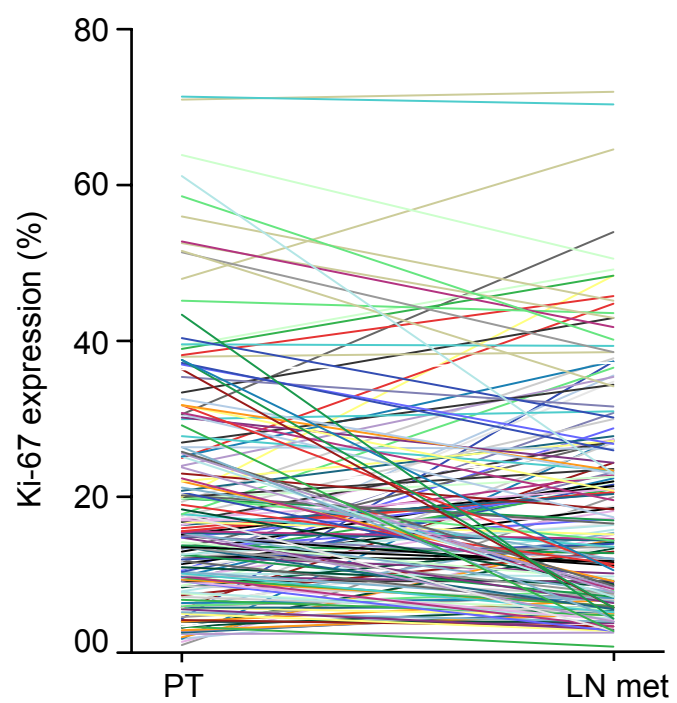

b.

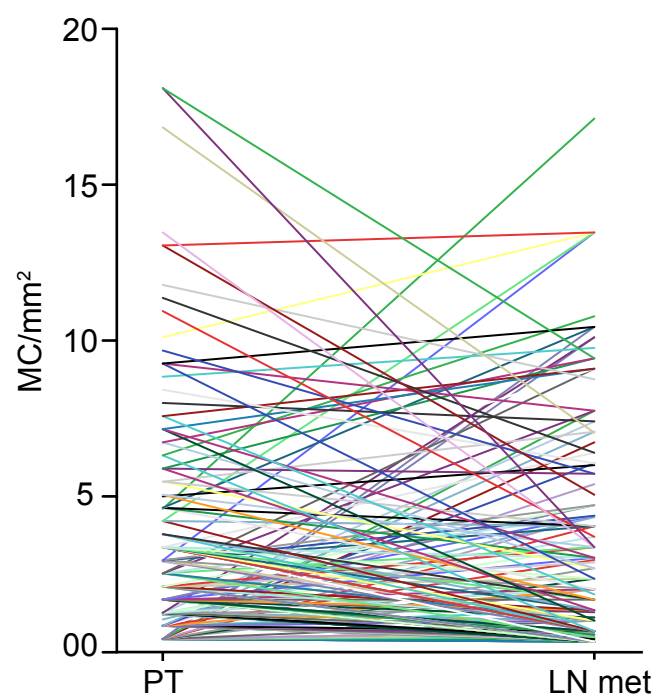

*Abbreviations:* PT; primary tumor, LN met; lymph node metastases

Supplement: S2 Fig — A parallel graphic illustration for the difference in ki-67 expression and mitotic count between PT and LN metastases. (PDF) [file pone.0150979.s002.pdf]

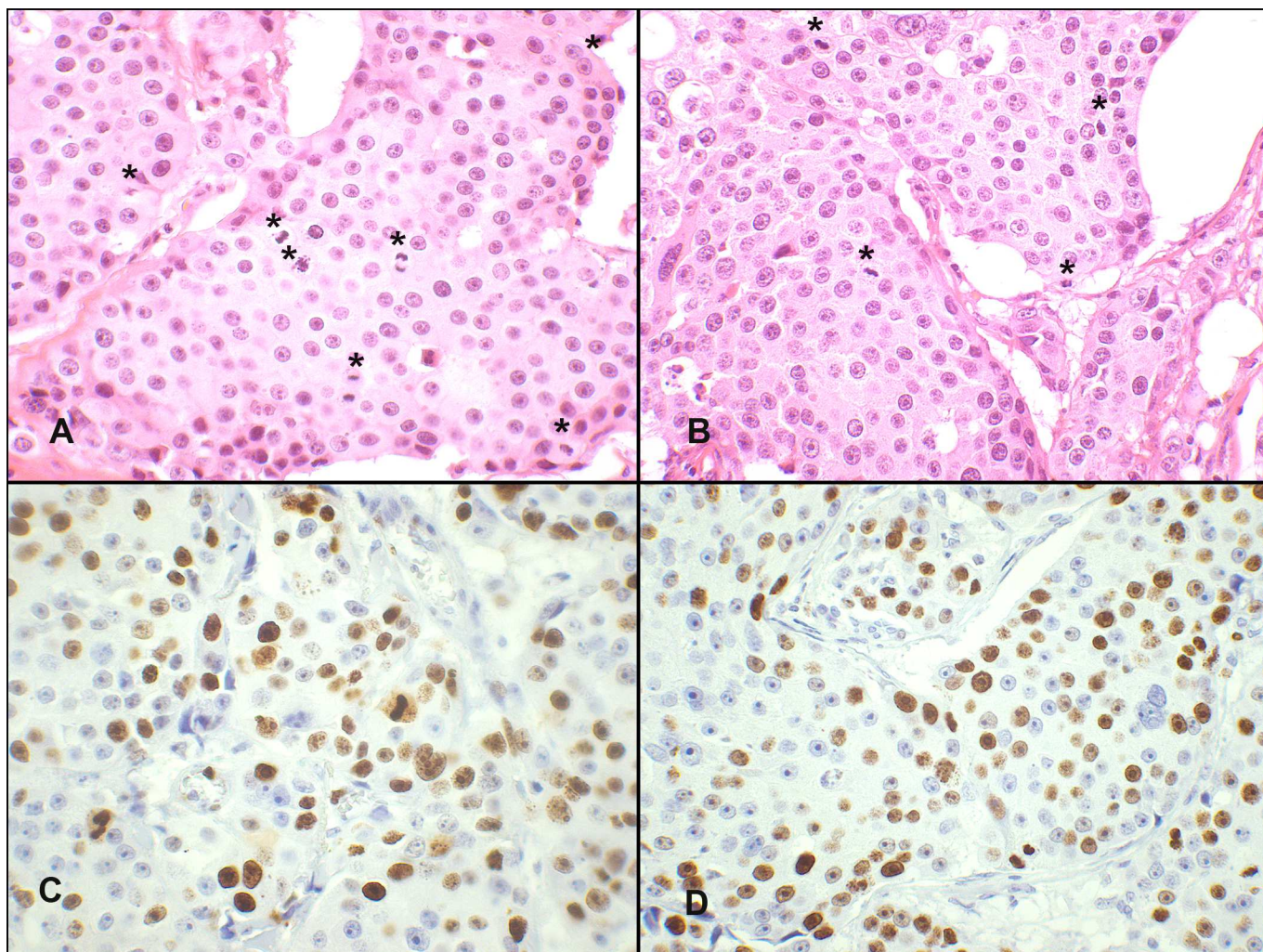

Supplement: S3 Fig — High mitotic count (asterisk) in PT (A) and LN (B). High Ki-67 expression in PT (C) and LN (D) (Leica, x 400). (PDF) [file pone.0150979.s003.pdf]

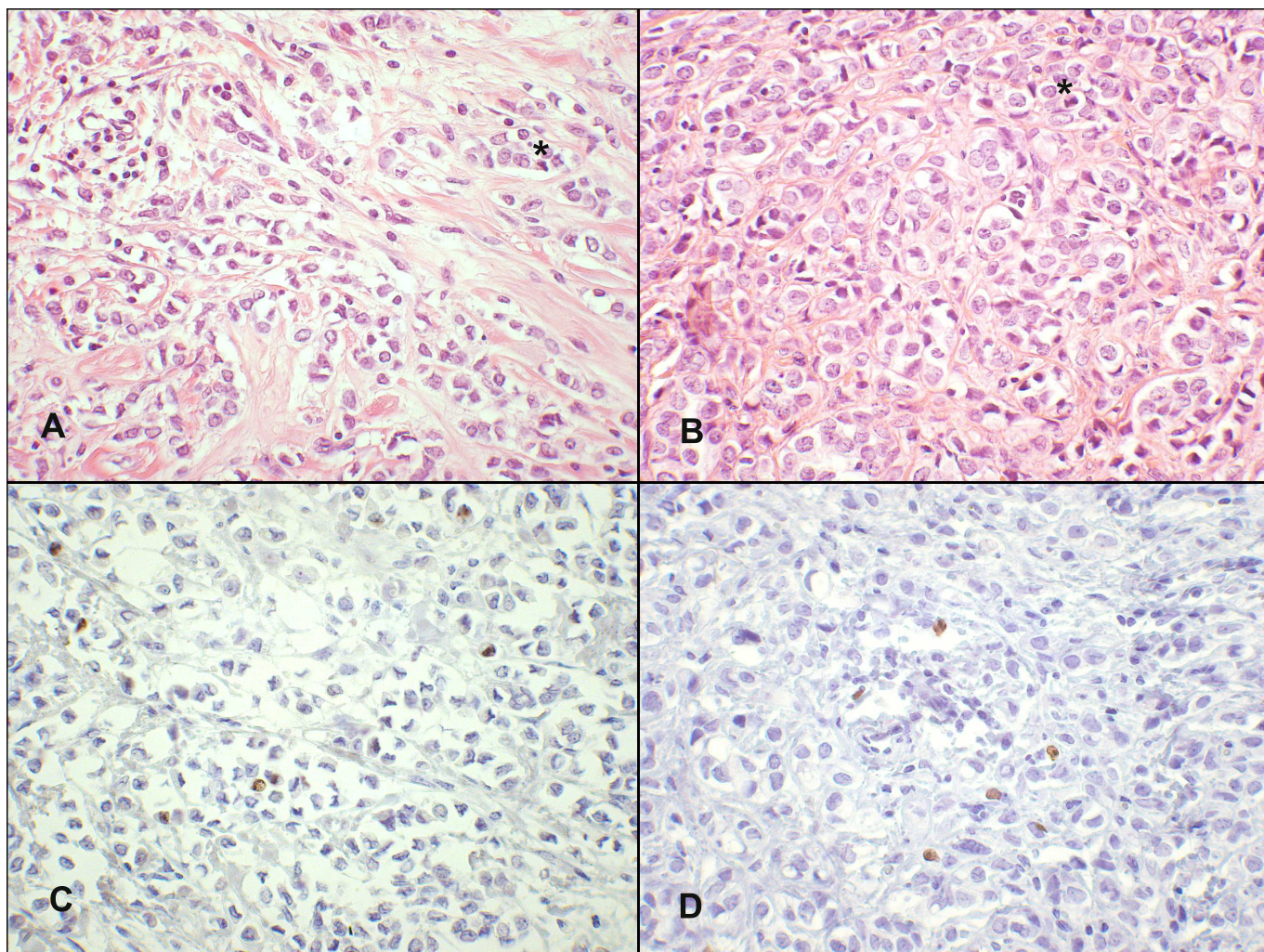

Supplement: S4 Fig — Low mitotic count (aterisk) in PT (A) and LN (B). Low Ki-67 expression in PT (C) and LN (D) (Leica, x 400). (PDF) [file pone.0150979.s004.pdf]

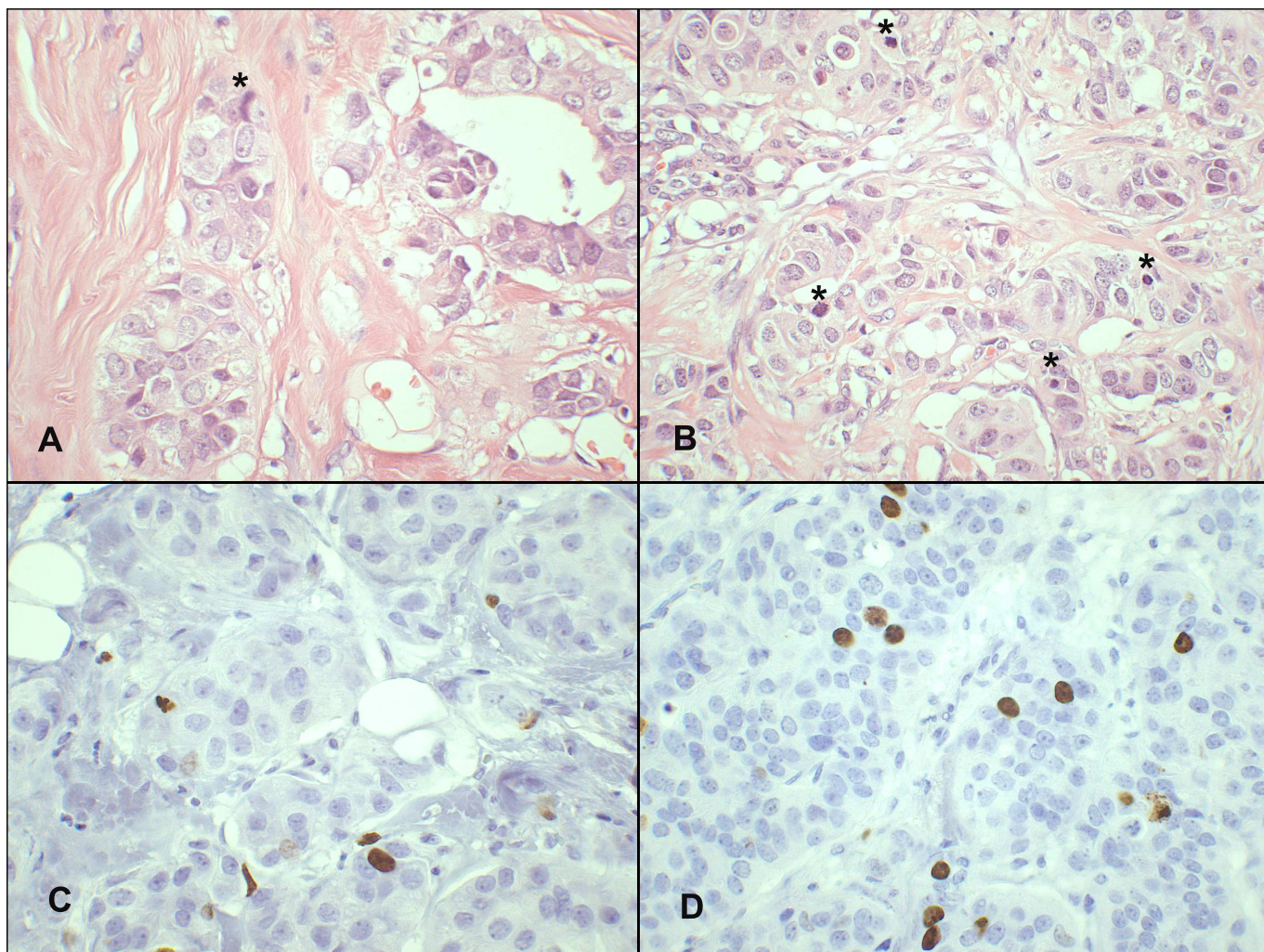

Supplement: S5 Fig — Low mitotic count (asterisk) and low Ki-67 expression in PT (A, C, respectively), while high mitotic count (asterisk) and high Ki-67 expression in LN (B, D, respectively) (Leica, x 400). (PDF) [file pone.0150979.s005.pdf]

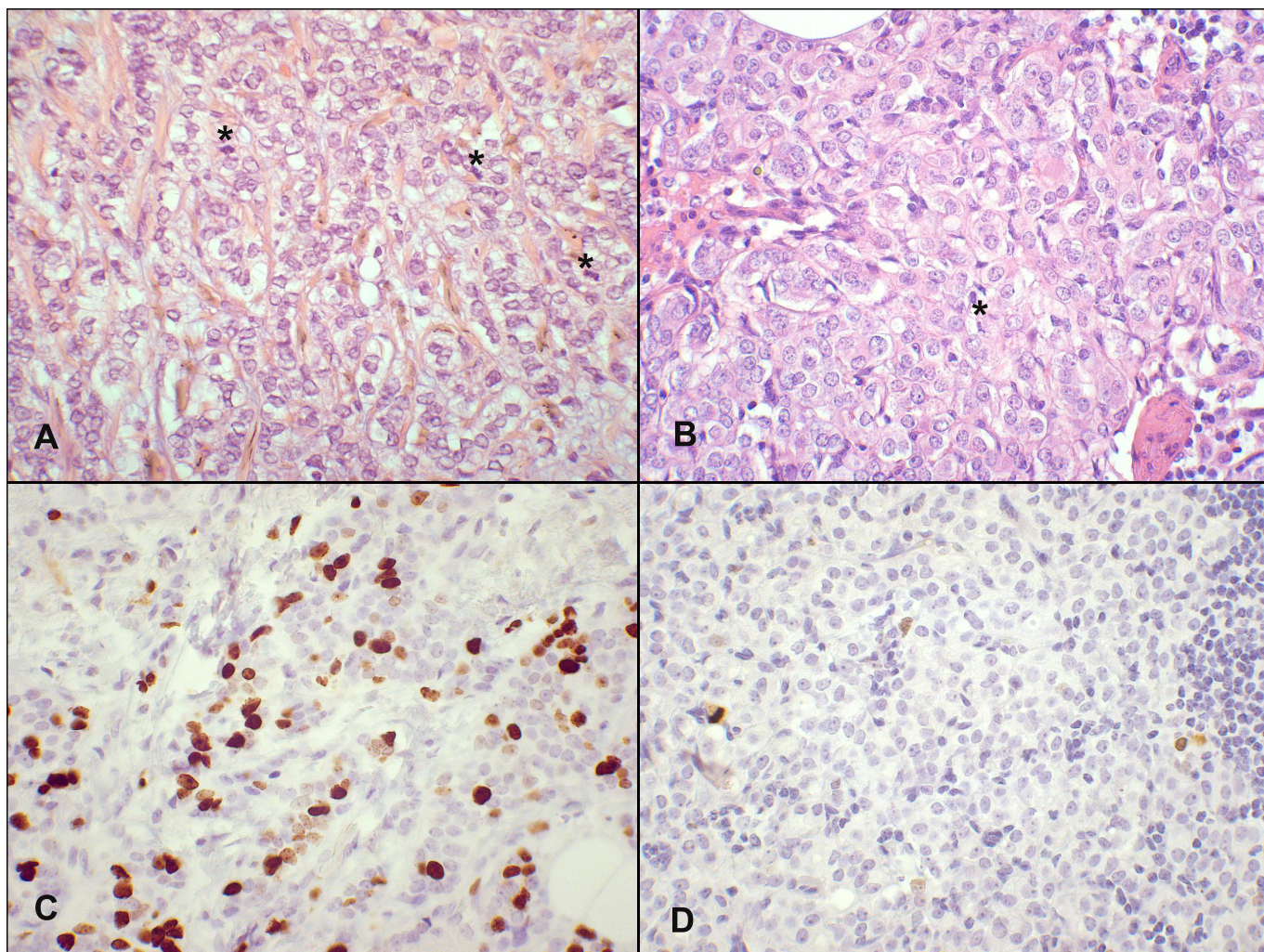

Supplement: S6 Fig — High mitotic count (asterisk) and high Ki-67 expression in PT (A, C, respectively), while low mitotic count (asterisk) and low Ki-67 expression in LN (B, D, respectively) (Leica, x 400). (PDF) [file pone.0150979.s006.pdf]

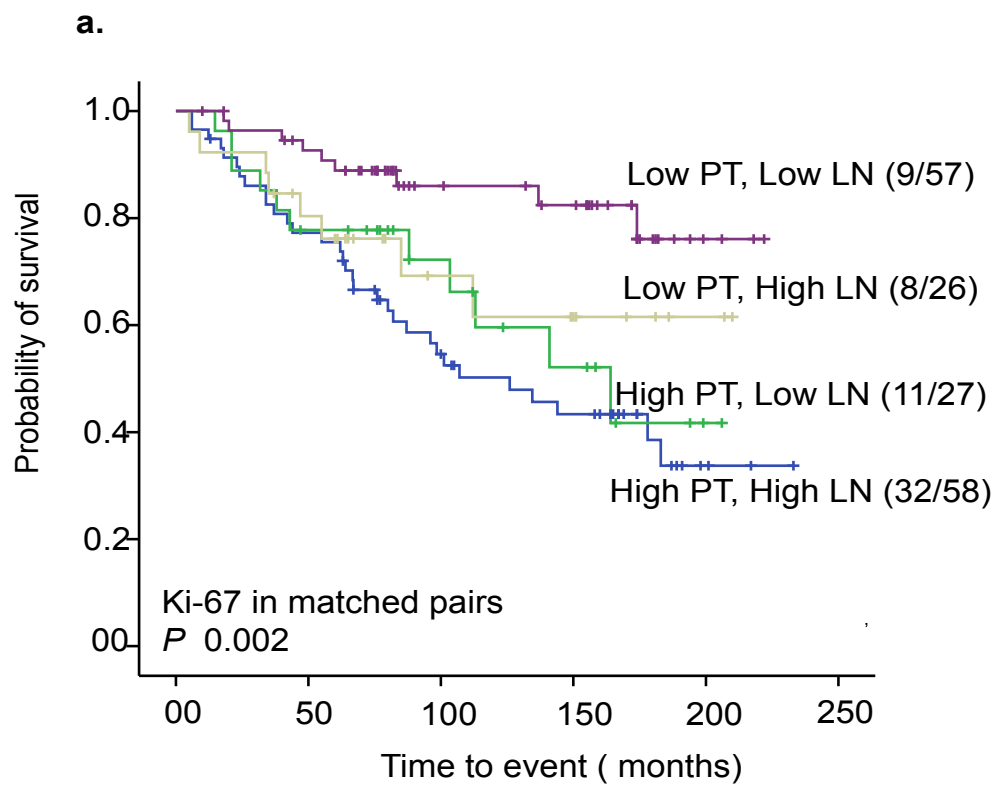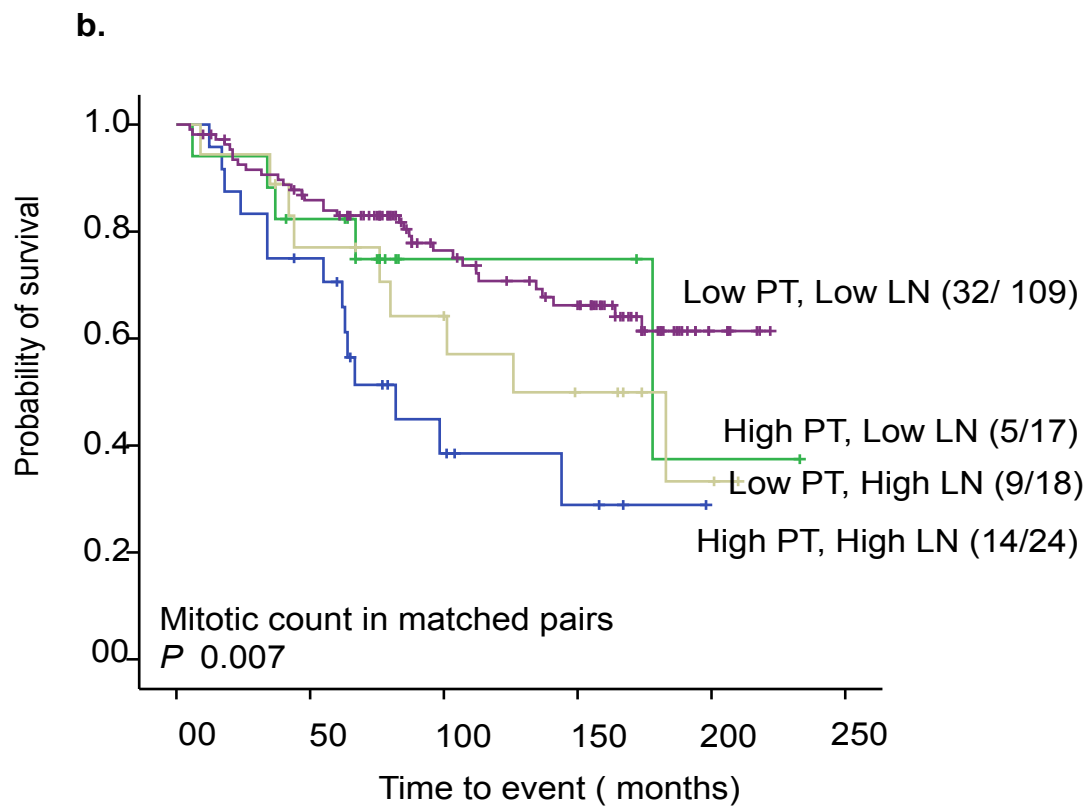

Supplement: S7 Fig — Survival curves (Kaplan-Meier method) are shown for Ki-67 (a) and for MC (b) in PT and LN metastasis in matched pairs. Number of events / number of cases are given in parenthesis. (PDF) [file pone.0150979.s007.pdf]
