# Supplementary material for: Evaluation of Tumor Cell Proliferation by Ki-67 Expression and Mitotic Count in Lymph Node Metastases from Breast Cancer
Source: PLoS One. 2016 Mar 8;11(3):e0150979. doi: 10.1371/journal.pone.0150979 (PMC4783103; doi:10.1371/journal.pone.0150979)
Supplement: S1 Table — Clinico-pathologic characteristics of primary tumors for the whole cohort before exclusion of some cases (n = 231). (DOCX) [file pone.0150979.s008.docx]

| **S1 Table** |  |  |  |  | |  | |  | | | |  |
| --- | --- | --- | --- | --- | --- | --- | --- | --- | --- | --- | --- | --- |
| **Clinico-pathologic characteristics of primary tumors in all lymph-node-positive cases (*n* = 231)** | | | | | | |  | |  | | |  |
|  |  |  |  |  | | |  | |  | | |  |
| **Variable** |  |  | **N** | | **(%)** |  | |  | | | |  |
| **Operation type** |  |  |  | |  |  | |  | | | |  |
| Mastectomy |  |  | 161 | | ( 69.6 ) |  | |  | | | |  |
| Breast conserving surgery |  |  | 69 | | ( 30.0 ) |  | |  | | | |  |
| Core needle biopsy ^a^ |  |  | 1 | | ( 0.4 ) |  | |  | | | |  |
| **Histologic type** |  |  |  | |  |  | |  | | | |  |
| Ductal carcinoma |  |  | 195 | | ( 84.4 ) |  | |  | | | |  |
| Lobular carcinoma |  |  | 30 | | ( 13.0 ) |  | |  | | | |  |
| Mucinøs carcinoma |  |  | 2 | | ( 0.9 ) |  | |  | | | |  |
| Undifferentiated |  |  | 2 | | ( 0.9 ) |  | |  | | | |  |
| Others |  |  | 2 | | ( 0.9 ) |  | |  | | | |  |
| **Histologic grade** |  |  |  | |  |  | |  | | | |  |
| Grade 1 |  |  | 63 | | ( 27.3 ) |  | |  | | | |  |
| Grade 2 |  |  | 116 | | ( 50.2 ) |  | |  | | | |  |
| Grade 3 |  |  | 52 | | ( 22.5 ) |  | |  | | | |  |
| **Tumor diameter (PT)** |  |  |  | |  |  | |  | | | |  |
| ≤ 2 cm |  |  | 132 | | ( 57.1 ) |  | |  | | | |  |
| ˃ 2 cm |  |  | 99 | | ( 42.9 ) |  | |  | | | |  |
| **Type of LN operation** |  |  |  | |  |  | |  | | | |  |
| SN and AXLN dissection |  |  | 101 | | ( 44.1 ) |  | |  | | | |  |
| Only axillary dissection |  |  | 122 | | ( 53.3 ) |  | |  | | | |  |
| Only SN biopsy |  |  | 6 | | ( 2.6 ) |  | |  | | | |  |
| Missing* |  |  | 2 | | (0.8) |  | |  | | | |  |
| **Positive nodes** |  |  |  | |  |  | |  | | | |  |
| 1-3 nodes |  |  | 168 | | ( 73.0) |  | |  | | | |  |
| ˃ 4 nodes |  |  | 62 | | ( 27.0 ) |  | |  | | | |  |
| Missing** |  |  | 1 | | ( 0.4 ) |  | |  | | | |  |
| **Type of metastasis ^b^** |  |  |  | |  |  | |  | | | |  |
| Micrometastasis |  |  | 42 | | ( 19.5 ) |  | |  | | | |  |
| Macrometastasis |  |  | 173 | | ( 80.5 ) |  | |  | | | |  |
| **ER status** |  |  |  | |  |  | |  | | | |  |
| Positive |  |  | 193 | | ( 83.5 ) |  | |  | | | |  |
| Negative |  |  | 38 | | ( 16.5 ) |  | |  | | | |  |
| **PR status** |  |  |  | |  |  | |  | | | |  |
| Positive |  |  | 154 | | ( 66.7 ) |  | |  | | | |  |
| Negative |  |  | 77 | | ( 33.3 ) |  | |  | | | |  |
| **Her2 status** |  |  |  | |  |  | |  | | | |  |
| Positive |  |  | 33 | | ( 14.3 ) |  | |  | | | |  |
| Negative |  |  | 197 | | ( 85.7) |  | |  | | | |  |
| Missing |  |  | 1 | | ( 0.4 ) |  | |  | | | |  |
| **Molecular subtypes ^c^** |  |  |  | |  |  | |  | | | |  |
| Luminal A |  |  | 91 | | ( 39.6 ) |  | |  | | | |  |
| Luminal B HER2 neg |  |  | 90 | | ( 39.1 ) |  | |  | | | |  |
| Luminal B HER2 pos |  |  | 18 | | ( 7.8 ) |  | |  | | | |  |
| HER2 positive |  |  | 15 | | ( 6.5 ) |  | |  | | | |  |
| Triple negative |  |  | 16 | | ( 7.0 ) |  | |  | | | |  |
| **Multifocal tumor (PT)** |  |  |  | |  |  | |  | | | |  |
| Yes |  |  | 35 | | ( 15.2 ) |  | |  | | | |  |
| No |  |  | 195 | | ( 84.8 ) |  | |  | | | |  |
| Missing |  |  | 1 | | ( 0.4 ) |  | |  | | | |  |
| **Distant metasasis** |  |  |  | |  |  | |  | | | |  |
| Yes |  |  | 79 | | ( 34.2 ) |  | |  | | | |  |
| No |  |  | 152 | | ( 65.8 ) |  | |  | | | |  |
| **Location of recurrence** |  |  |  | |  |  | |  | | | |  |
| Skeletal |  |  | 49 | | ( 21.2 ) |  | |  | | | |  |
| Lung |  |  | 27 | | ( 11.7 ) |  | |  | | | |  |
| Liver |  |  | 43 | | ( 18.6 ) |  | |  | | | |  |
| Brain |  |  | 18 | | ( 7.8 ) |  | |  | | | |  |
| Pleura |  |  | 9 | | ( 3.9 ) |  | |  | | | |  |
| Axilla |  |  | 5 | | ( 2.0 ) |  | |  | | | |  |
| Skin |  |  | 3 | | ( 1.3 ) |  | |  | | | |  |
| Adrenal gland |  |  | 1 | | ( 0.4 ) |  | |  | | | |  |
| Peritoneum |  |  | 3 | | ( 1.3 ) |  | |  | | | |  |
| **Status** |  |  |  | |  |  | |  | | | |  |
| Alive |  |  | 131 | | ( 56.7 ) |  | |  | | | |  |
| Alive with metastasis |  |  | 34 | | ( 14.7 ) |  | |  | | | |  |
| Death breast cancer |  |  | 48 | | ( 20.8 ) |  | |  | | | |  |
| Death other reasons |  |  | 18 | | ( 7.8 ) |  | |  | | | |  |
|  |  |  |  | |  |  | |  | | | |  |
| ^a^ Core Needle Biopsy was performed on PT and LN in one case because of locally advanced cancer disease with deteriorated clinical condition  ^b^ Missing cases: 10 cases with missing tumor tissue, 6 cases with FNAC. Type of lymph node metastasis ( micrometastasis < 2 mm, macrometastasis ≥ 2 mm) | | | | | | | | | |  |  | |
| ^c^ Hormonal positivity was determined as positivity for ER and /or PR (St.Gallen 2015).  *Two cases were missing with respect to the type of lymph node removal procedure; one  With CNB and the other with FNAC from unspecified lymph node.  ** One case with no available information about the number of positive nodes because of locally  Advanced cancer with conglomerated metastatic lymph nodes. | | | | | | | | | | | | |
|  | | | | | | | | | |  |  | |
|  |  |  |  | |  |  | |  | | | |  |
|  |  |  |  | |  |  | |  | | | |  |
|  |  |  |  | |  |  | |  | | | |  |
