# Supplementary material for: Evaluation of Tumor Cell Proliferation by Ki-67 Expression and Mitotic Count in Lymph Node Metastases from Breast Cancer
Source: PLoS One. 2016 Mar 8;11(3):e0150979. doi: 10.1371/journal.pone.0150979 (PMC4783103; doi:10.1371/journal.pone.0150979)
Supplement: S2 Table — Clinico-pathologic characteristics of primary tumors for the cases which were finally included in this study (n = 168). (DOCX) [file pone.0150979.s009.docx]

| **S2 Table** |  |  |  | |  |  |  |
| --- | --- | --- | --- | --- | --- | --- | --- |
| **Clinico-pathologic characteristics of the primary tumors in lymph-node positive cases** | | | | | | | |
| **with metastases ≥ 2.0 mm ( *n* = 168 )** | |  |  | |  |  |  |
| **Characteristics** |  | **N** | **%** | |  |  |  |
| **Tumor diameter ᵅ** |  |  |  | |  |  |  |
| ≤ 2 cm |  | 95 | 56.5 | |  |  |  |
| >2 cm |  | 73 | 43.5 | |  |  |  |
| **Histologic type** |  |  |  | |  |  |  |
| Ductal |  | 145 | 86.3 | |  |  |  |
| Lobular |  | 19 | 11.3 | |  |  |  |
| Mucinous |  | 2 | 1.2 | |  |  |  |
| Others |  | 2 | 1.2 | |  |  |  |
| **Histologic grade** |  |  |  | |  |  |  |
| Grade 1 |  | 51 | 30.4 | |  |  |  |
| Grade 2 |  | 78 | 46.4 | |  |  |  |
| Grade 3 |  | 39 | 23.2 | |  |  |  |
| **MC/mm² ᵇ** |  |  |  | |  |  |  |
| ≤ 4.2 |  | 127 | 75.6 | |  |  |  |
| ˃ 4.2 |  | 41 | 24.4 | |  |  |  |
| **ER** |  |  |  | |  |  |  |
| Positive |  | 141 | 83.9 | |  |  |  |
| Negative |  | 27 | 16.1 | |  |  |  |
| **PR** |  |  |  | |  |  |  |
| Positive |  | 115 | 68.5 | |  |  |  |
| Negative |  | 53 | 31.5 | |  |  |  |
| **HER2** |  |  |  | |  |  |  |
| Negative |  | 143 | 85.1 | |  |  |  |
| Positive |  | 25 | 14.9 | |  |  |  |
| **Ki-67 (%) ᵅ** |  |  |  | |  |  |  |
| ≤ 14.2 |  | 83 | 49.4 | |  |  |  |
| ˃ 14.2 |  | 85 | 50.6 | |  |  |  |
| **Molecular subtypes ᶜ^, d^** |  |  |  | |  |  |  |
| Luminal A |  | 62 | 36.9 | |  |  |  |
| Luminal B/ HER2 - |  | 69 | 41.1 | |  |  |  |
| Luminal B/ HER2+ |  | 15 | 8.9 | |  |  |  |
| HER2 + |  | 11 | 6.5 | |  |  |  |
| Triple negative |  | 11 | 6.5 | |  |  |  |
| **No. of involved lymph nodes ^e^** |  |  |  | |  |  |  |
| 1-3 nodes |  | 107 | 64.1 | |  |  |  |
| ≥ 4 nodes |  | 60 | 35.9 | |  |  |  |
| **Distant metastasis** |  |  |  | |  |  |  |
| No |  | 108 | 64.3 | |  |  |  |
| Yes |  | 60 | 35.7 | |  |  |  |
| N, number of cases in each subgroup; MC, mitotic count; ER, estrogen receptor; | | | | | | | |
| PR, progesterone receptor |  |  | |  |  |  |  |
| ᵅ Cut-off value at median |  |  | |  |  |  |  |
| ᵇ Cut-off value at upper quartile |  |  | |  |  |  |  |
| ᶜ Molecular subtype according to St.Gallen 2013  ^d^ Determining hormonal receptor status was dependent on positivity of ER and/or PR. | | | |  |  |  |  |
| ^e^ One case is missing regarding number of metastatic lymph nodes because of fused metastatic lymph nodes in a patient with locally advanced disease | | | | | |  |  |
